# Role of Fish Oil in Preventing Paternal Obesity and Improving Offspring Skeletal Muscle Health

**DOI:** 10.3390/biomedicines11123120

**Published:** 2023-11-23

**Authors:** Ligeng Xiong, Stephen Dorus, Latha Ramalingam

**Affiliations:** 1 Department of Nutrition and Food Studies, Syracuse University, Syracuse, NY 13244, USA; 2 Department of Biology, Syracuse University, Syracuse, NY 13244, USA

**Keywords:** epigenetic, intergenerational, omega-3-fatty acids

## Abstract

This study investigates the effects of fish oil supplementation during the periconceptional period in male mice. Specifically, it examines the impact of fish oil on intergenerational health, as determined by skeletal muscle markers. To mimic paternal obesity, thirty mice were separated into three groups with distinct dietary regimes for 10 weeks: a high-fat diet (HF), a high-fat diet supplemented with fish oil (FO), and a low-fat diet (LF). Then, these mice mated with control female mice. Dams and offspring consumed a chow diet during gestation and lactation, and the offspring continued on a chow diet. To study short-term (8 weeks) and long-term (16 weeks) effects of FO, skeletal muscle was isolated at the time of sacrifice, and gene analyses were performed. Results suggest that offspring born to FO-supplemented sires exhibited a significant, short-term upregulation of genes associated with insulin signaling, fatty acid oxidation, and skeletal muscle growth with significant downregulation of genes involved in fatty acid synthesis at 8 weeks. Prominent differences in the above markers were observed at 8 weeks compared to 16 weeks. These findings suggest the potential benefits of FO supplementation for fathers during the periconceptional period in reducing the health risks of offspring due to paternal obesity.

## 1. Introduction

Broadly, obesity rates have increased from 30.5% in 2000 to 42.5% in 2020 [1]. Obesity, defined as excess accumulation of adipose tissue, is associated with an increased secretion of proinflammatory adipokines. Moreover, increased energy intake is also linked to excess production of free radicals [2,3]. Both these pathological stresses contribute to comorbidities of obesity including type II diabetes (T2D) and cardiovascular disorders. 

Dysfunction in the skeletal muscle contributes to insulin resistance and T2D [4], which, in turn leads to obesity. Increased production of reactive oxygen species (ROS) leads to muscle atrophy and contractile malfunction, eventually developing into sarcopenia and skeletal muscle dysfunction [5,6,7,8,9]. Additionally, inflammation also contributes to higher ROS production and lipid peroxidation, which further contributes to impairment of skeletal muscle function [4]. All these factors combined decrease mitochondrial biogenesis, resulting in reduced mitochondrial numbers and size, which affects the skeletal muscle [10,11,12,13,14]. Lastly, lipid infiltrates from adipose tissue into muscle, thereby limiting muscle synthesis and insulin sensitivity [15].

Behavioral and surgical interventions have been extensively studied; however, research on dietary supplement interventions ameliorating complex obesity-associated conditions remains limited. In this study, we focused on fish oil (FO), which contains two polyunsaturated fatty acids (PUFAs): docosahexaenoic acid (DHA) and eicosapentaenoic acid (EPA). FO has numerous benefits in skeletal muscle function through increased utilization of fatty acids, improved skeletal muscle recovery, and inhibition of inflammation [16]. Additionally, FO reduces intracellular lipid levels by increasing mitochondrial biogenesis and fatty acid oxidation via the Ppara pathway [17]. In addition to these beneficial effects, FO has several advantages in maternal obesity and thereby improves metabolic health of the offspring. 

Several studies have revealed intergenerational consequences of maternal obesity on offspring health [18,19,20,21], but there exists limited focus on paternal effects. Paternal obesity contributes to increased ROS production in sperm, impairs sperm quality, and potentially affects future offspring [22]. Further, inflammatory conditions can have a detrimental impact on the metabolic health of spermatozoa (and oocytes), and these effects may persist into embryonic development and beyond [23]. For example, paternal obesity influences offspring birth weight as well as contributes to abnormal muscle and fat development [24]. Paternal exercise has also been shown to reduce skeletal muscle insulin resistance in offspring when combined with a high-fat diet [25].

FO supplementation during pregnancy has shown to improve the health outcomes of mice offspring in terms of weight and insulin sensitivity [26,27,28]. However, the potential beneficial role of FO supplementation in obese fathers and offspring metabolic health has not been investigated. In this study, we focus specifically on the effect of paternal FO supplementation on offspring skeletal muscle function, including the expression of genes associated with skeletal muscle function. 

## 2. Materials and Methods

Animal studies: Thirty C57BL6J male and female mice, aged 4–5 weeks, were purchased from Jackson Labs (Bar Harbor, ME, USA). Male mice were divided into three groups with three different dietary interventions: 8 mice were fed a low-fat (LF) diet containing 13% fat, 28% protein, and 58% carbohydrates. Eleven mice were fed a high-fat (HF) diet containing 45% fat, 28% protein, and 27% carbohydrates, and 11 mice were fed an HF diet supplemented with fish oil (HF-FO) containing 45% fat with fish oil, 28% proteins, and 27% carbohydrates along with 30 gm/FO for 10 weeks. Fish oil isolated from ocean fish (sardines, anchovies, and herring) was a kind gift from DSM (Parsippany, NJ, USA) and contained ~720 mg/g of EPA as ethyl esters. Total omega-3s measured as ethyl esters were ~770 mg/g. Diets were purchased from Research Diets (New Brunswick, NJ, USA). 

Food intake and body weight of the mice were recorded weekly. After 10 weeks, male mice were mated with 10-week-old female mice on a chow diet. All female mice were fed a chow diet during the mating period. Once conception was confirmed, pregnant mice were separated from the male mice and fed a chow diet during gestation and lactation. Offspring mice were weaned at three weeks. All protocols were approved by the Institutional Animal Care and Use committee of Syracuse University. Out of 217 offspring born to all the dams, only 173 survived as shown in Table 1.

Body weight and food consumption were recorded weekly. Offspring were separated into short-term and long-term groups and sacrificed at 8 weeks and 16 weeks, respectively. Following weaning, mice continued on a chow diet until sacrifice. Mice were euthanized using isoflurane, and gastrocnemius muscle was isolated; tissues were stored at −80 °C until further analyses. 

Insulin tolerance tests (ITT): At 10 weeks of dietary intervention, ITTs were administered. Basal blood glucose was measured using a handheld glucometer (Abbott Laboratories, Alameda, CA, USA) following a five-hour fast. Mice were then injected with 1 IU insulin/kg (Eli Lilly, Indianapolis, IN, USA); glucose measurements were taken at 15, 30, 45, 60, and 90 min, respectively.

RNA isolation: Gastrocnemius muscle was homogenized with Trizol Solution (Ambion by Life Technologies, Carlsbad, CA, USA), using Tissue Lyzer (Qiagen, Hilden, Germany). RNA was extracted using Zymo Research Quick-RNA MicroPrep Kit (Zymo Research, Irvine, CA, USA). Complementary DNA (cDNA) was synthesized from the extracted RNA using the Applied biosystems High-Capacity cDNA Reverse Transcription Kit (Thermo Fisher, San Jose, CA, USA). Gene expression was analyzed using Sybr (Thermo Fisher, San Jose, CA, USA) and the QuantstudioTM 3 Real-Time PCR System. The qPCR results were calculated in the quant studio software provided by applied biosystems. Relative gene expression of target genes was normalized using the HF group as the control and actin as the housekeeping gene, and the relative normalized expression was calculated using the delta delta comparative threshold method (ΔΔCt). Primer sequences are provided in Table 2.

Statistical analysis: One-way ANOVA followed with Tukey’s post-hoc test was performed using GraphPad Prism version 9.0.0 for Windows, GraphPad Software, Boston, MA, USA, www.graphpad.com. Two-way ANOVA was used to test for sex, diet, and time effects. Significance was considered at a 95% confidence with *p* < 0.05. 

## 3. Results

### 3.1. Offspring Father Body Weight

**Figure 1:**
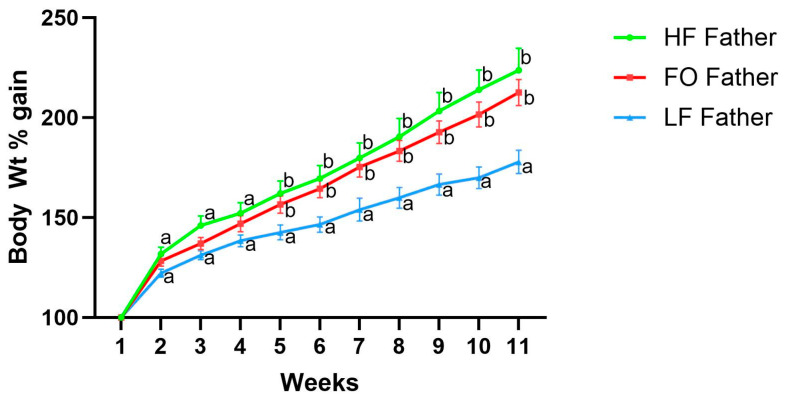
Body weight percentage gain of fathers with HF, FO, and LF supplementation. Common letters on the error bars indicate no significance (e.g., “a” is significantly different from “b”, and “ab” indicates no significance compared to “a” and “b”).

All male mice were randomized into three different groups and had no difference in body weight in the beginning. Further, all father groups were comparable until 4 weeks (Figure 1). Male mice fed HF gained significantly higher body weight compared to LF starting from week 5 to week 11. Additionally, no significant change was observed between body weight in HF fathers and FO fathers. 

### 3.2. Offspring Development and Body Weight

**Figure 2:**
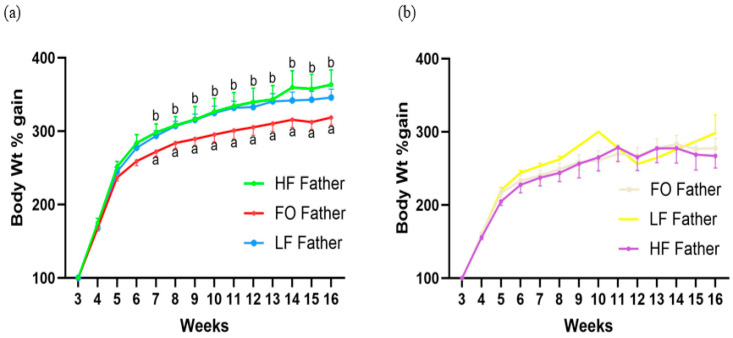
Body weight of male (**a**) and female (**b**) offspring born to fathers with LF, HF, and FO. Body weight is represented as weight gain in percentage from the weaning weight at 3 weeks. Common letters on the error bars indicate no significance (e.g., “a” is significantly different from “b”).

Male offspring mice had a statistically indistinguishable weight gain between 3–6 weeks (Figure 2a). Offspring born to FO fathers had a significantly reduced weight gain beginning at 7 weeks and a statistically lower final weight at 16 weeks. In contrast, offspring born to HF fathers gained similar body weight as offspring born to LF fathers. Lastly, no significant difference was observed in the weight gain or final weight of female offspring across treatments, as shown in Figure 2b.

### 3.3. Offspring Insulin Resistance

**Figure 3:**
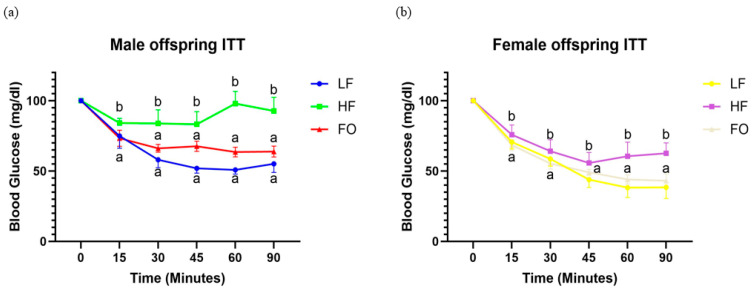
Insulin tolerance test for male (**a**) and female (**b**) offspring born to HF, FO, and LF fathers. Common letters on the error bars indicate no significance (e.g., “a” is significantly different from “b”).

Male and female offspring born to LF fathers, as expected, exhibited reduced blood glucose values at all time points after insulin injection (Figure 3a,b). Additionally, both male and female offspring born to HF fathers exhibited significantly higher blood glucose levels at 15, 30, 45, 60, and 90 min compared to the offspring born to LF fathers, consistent with insulin resistance. In contrast, both male and female offspring mice born to fathers in the FO supplement treatment had significantly lower blood glucose levels than offspring born to HF fathers (Figure 3a,b), indicative of enhanced insulin sensitivity.

### 3.4. Gene Expression Related to Fatty Acid Oxidation

We aimed to investigate the genetic mechanisms underlying the observed differences in ITTs, with a specific focus on skeletal muscle, which is responsible for 80% of glucose uptake. An increased rate of beta oxidation is beneficial for skeletal muscle to promote better insulin sensitivity [29,30,31]. Hence, we quantified the expression of four markers of fatty acid oxidation. Male offspring of FO-fed fathers exhibited significantly higher expression in all the four markers compared to offspring born to HF-fed fathers, with three of the four genes showing higher fatty acid oxidation in females. As indicated in Table 3, female offspring had a higher relative expression for each gene compared to male offspring, except *Acat*. 

Also, multiple comparisons between the diets are provided in supplemental Table S1. Further, we measured these markers at two points, 8 and 16 weeks, respectively, to study short- and long-term effects of paternal FO supplementation in the offspring. Interestingly, prominent improvements with FO were found for fatty acid oxidation markers at 8 weeks, with substantially less widespread differences at 16 weeks (supplementary data). Hence, we focused on sex differences at 8 weeks.

**Figure 4:**
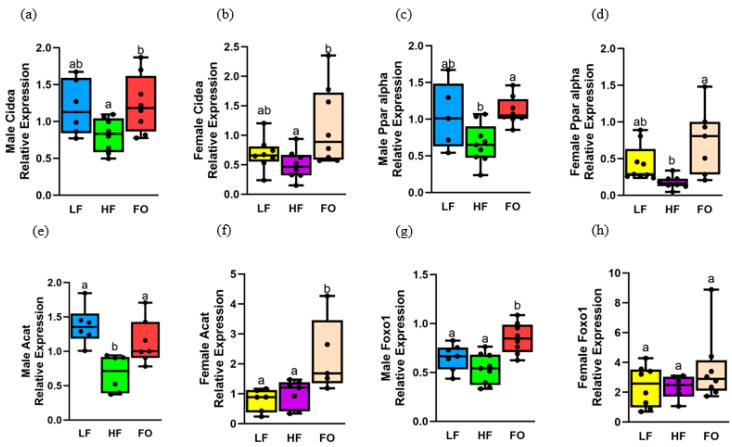
Gene expression of markers related to fatty acid oxidation in muscle of 8-week male and female mice. Relative normalized expression of (**a**,**b**) cell death-inducing DNA fragmentation factor (Cidea) in males and females, respectively, (**c**,**d**) peroxisome proliferator-activated receptor alpha (Ppar alpha) in males and females, respectively, (**e**,**f**) acetyl-CoA acetyltransferase (Acat) in males and females, respectively, and (**g**,**h**) forkhead box protein O1 in males and females, respectively (Foxo1). Common letters on the error bars indicate no significance (e.g., “a” is significantly different from “b”, and “ab” indicates no significance compared to “a” and “b”).

Expression of cell death-inducing DNA fragmentation alpha-like effector A (*Cidea*) and peroxisome proliferator-activated receptor alpha (*Ppara*) was comparable between HF and LF groups in both 8-week male and female offspring (Figure 4a–d). However, FO significantly increased *Cidea* and *Ppara* levels in both female and male offspring compared to offspring of HF fathers, as shown in Figure 4a–d. Additionally, diet and sex differences were found for both markers, as shown in Table 3; *Cidea* levels were higher in male offspring born to fathers fed HF compared to their counterpart females (Supplemental Table S1), while both LF and HF male offspring had higher levels of *Ppara* compared to the respective female offspring groups.

Another fatty acid oxidation marker, acetyl-CoA acetyltransferase (*Acat*), was lower in HF compared to LF male offspring but comparable between HF and LF females (Figure 4e,f). However, FO significantly increased *Acat* levels in both male and female offspring compared to offspring of HF fathers. Diet differences and a diet–sex interaction were observed for *Acat*. Lastly, forkhead box protein O1 (*Foxo1*) levels were similar in both LF and HF males and females, as shown in Figure 4g,h. However, FO significantly increased *Foxo1* in 8-week male offspring compared to both LF and HF, as demonstrated in Figure 4g, with no changes with FO in female offspring. None of these markers were altered in male mice at 16 weeks (Supplementary Figure S1a–d).

### 3.5. Gene Expression Related to Fatty Acid Synthesis

**Figure 5:**
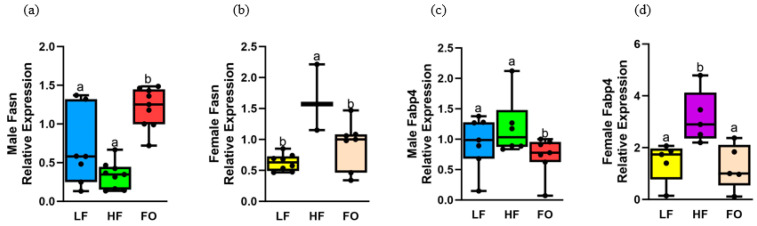
Gene expression of markers related to fatty acid synthesis in muscle of 8-week male and female mice. (**a**,**b**) Fatty acid synthase (Fasn) in males and females, respectively, (**c**,**d**) fatty acid binding protein 4 (Fabp4) in males and females, respectively. Common letters on the error bars indicate no significance (e.g., “a” is significantly different from “b”).

As we observed improvements in markers of fatty acid oxidation with FO, another pathway that could be altered along with fatty acid oxidation is synthesis of fatty acids. High-fat diet-induced obesity leads to higher fatty acid synthesis with intramyocellular lipid build up and reduced insulin sensitivity [32]. Hence, we measured two markers of fatty acid synthesis: fatty acid synthase (*Fasn*) and fatty acid binding protein (*Fabp4)*. Both these markers were higher with HF diet-induced obesity in females but not in males, but FO reduced fatty acid synthesis for one marker in males and for both markers in females. For *Fasn*, no difference was found between LF and HF male offspring (Figure 5a). However, FO significantly increased *Fasn* levels in male offspring compared to HF. Further, *Fasn* levels in HF female offspring were significantly higher compared to LF female offspring, and FO significantly decreased it in female offspring compared to HF (Figure 5b). We found diet and sex differences, as well as a diet–sex interaction. Female offspring had higher *Fasn* levels than males (Supplemental Table S1). Another fatty acid synthesis marker, *Fabp4* levels, were comparable between HF and LF mice in male offspring, as shown in Figure 5c. However, *Fabp4* levels were higher in HF compared to LF female offspring (Figure 5d). Further, *Fabp4* levels were downregulated with FO in both male and female offspring compared to HF. Diet and sex differences were found. Lastly, no changes in these markers were observed in 16-week male mice (Supplemental Figure S1e,f)

### 3.6. Gene Expression Relating to Insulin Signaling

Obesity negatively affects insulin signaling in obese mice [33,34], as evidenced by insulin-signaling markers, including glucose transporter type 4 (*Glut4*) and insulin-receptor substrate 1 (*Irs1*), which are downregulated with HF exposure [21,35]. In our study, we measured expression of important insulin signaling markers: *Irs1*, *Pi3k,* and *Glut4*. We found that all markers measured were upregulated with FO compared to HF, with female mice having a higher relative expression of all markers compared to males.

**Figure 6:**
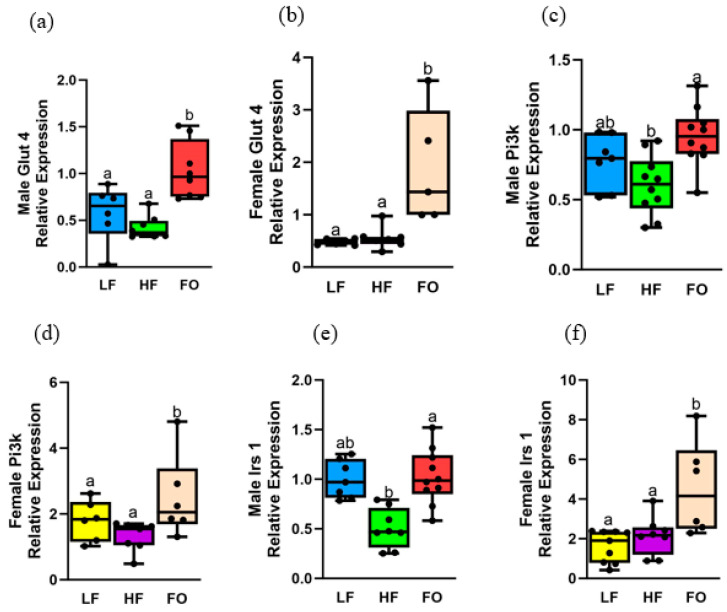
Gene expression of markers related to insulin signaling in muscle of 8-week male and female mice. Relative normalized expression of (**a**,**b**) glucose transporter type 4 (Glut4) in males and females, respectively, (**c**,**d**) phosphatidylinositol-3 kinase (Pi3k) in males and females, respectively, (**e**,**f**) insulin substrate 1 (Irs1) in males and females, respectively. Groups with the same letter indicate no statistical significance, and groups with different letters indicate significance at *p* value less than 0.05.

*Glut4* and *Pi3k* showed comparable levels between LF and HF in both female and male offspring groups at 8 weeks (Figure 6a–d). Interestingly, FO significantly increased both *Glut4* and *Pi3k* levels in both 8-week male and female offspring compared to both HF and LF. Diet- and sex differences were observed for *Glut4* and *Pi3k*, as well as a diet–sex interaction for *Glut4* but not for *Pi3k*. Female offspring from FO fathers had significantly higher Glut4 levels compared to male offspring of FO fathers (Supplemental Table S1), while all female offspring had significantly higher expression of *Pi3k* in all dietary groups compared to male offspring. 

We then measured *Irs1* levels, which were comparable between HF and LF in male offspring but were significantly increased with FO (Figure 6e). Interestingly, FO increased *Irs1* levels in female offspring compared to both LF and HF fathers (Figure 6f). Both diet and sex difference were found, with an interaction between them: higher *Irs1* levels were found in female offspring of the FO group compared to their male counterparts. *Glut4* and *Irs1* were not altered in 16-week muscle, but FO increased *Pi3k* levels compared to HF (Supplemental Figure S2a–c).

### 3.7. Gene Expression Relating to Mitochondrial Oxidation

**Figure 7:**
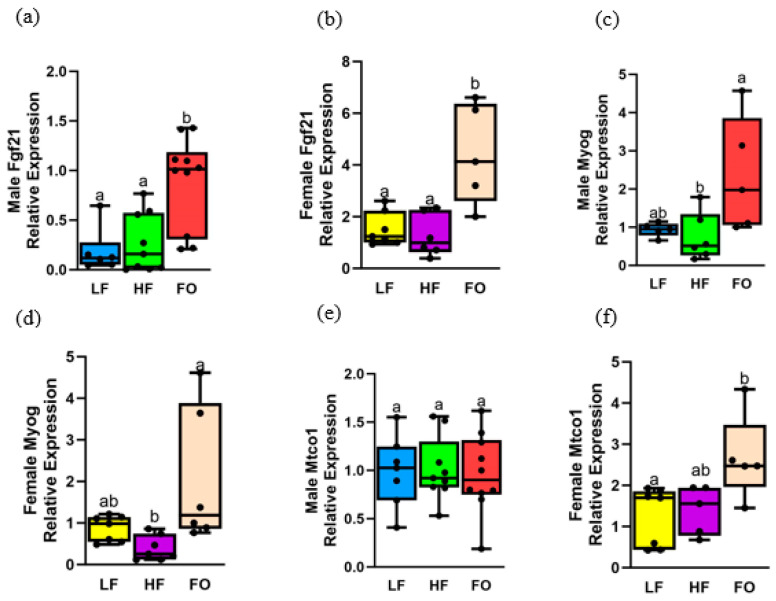
Gene expression of markers related to mitochondrial oxidation in muscle of 8-week male and female mice. Relative normalized expression of (**a**,**b**) fibroblast growth factor 21 (Fgf21) in males and females, respectively, (**c**,**d**) myogenin (Myog) in males and females, respectively, and (**e**,**f**) mitochondrially encoded cytochrome c oxidase I (Mtco1) in males and females, respectively. Groups with the same letters indicate no statistical significance, and groups with different letters indicate significance at *p* value less than 0.05.

Mitochondrial oxidation is another pathway that is altered in muscle with obesity [10], leading to mitochondrial dysfunction. When myoblasts undergo differentiation to form myotubes, there is a significant rise in the activity of these mitochondrial enzymes. Hence, we measured three markers involved in mitogenesis and oxidation. Out of the three genes measured, FO significantly upregulated two of the three markers in males, but all markers were upregulated by FO in females. Sex differences were depicted, with female offspring overall having a higher expression compared to male offspring in three of four genes measured. As indicated in Figure 7a–f, LF and HF groups in both female and male offspring had similar levels of all markers measured: fibroblast growth factor 21 (*Fgf21*), myogenin (*Myog*) levels, and mitochondrially encoded cytochrome c oxidase I (*Mtco1*). However, FO increased *Fgf21* and *Myog* levels in both male and female offspring compared to other groups (Figure 7a–d). FO increased *Mtco1* in females but not in males. Diet and sex differences, along with a diet and sex interaction, were found. Female offspring of an HF father had a higher expression of *Fgf21* compared to male offspring (Supplemental Table S1). For *Myog*, a significant difference was found with diet only. Diet and sex interaction was found with differences in both diet and sex for *Mtco1*. At 16 weeks, FO significantly increased *Fgf21* and *Myog* but not *Mtco1*(Supplemental Figure S3a–c).

### 3.8. Gene Expression Related to Oxidative Stress

**Figure 8:**
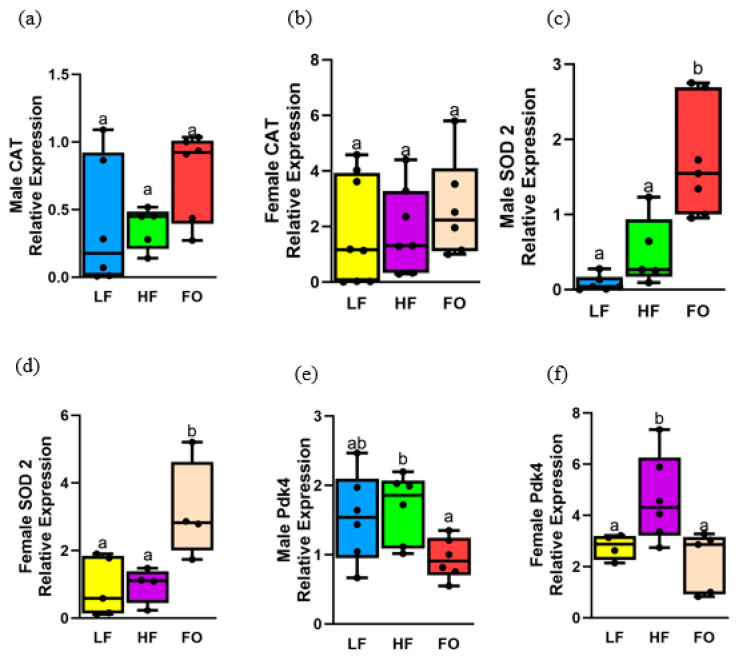
Gene expression of markers related to oxidative stress in muscle of 8-week male and female mice. Relative normalized expression of (**a**,**b**) catalase (CAT) in males and females, respectively, (**c**,**d**) superoxide dismutase 2 (Sod2) in males and females, respectively, and (**e**,**f**) pyruvate dehydrogenase kinase 4 (Pdk4) in males and females, respectively. Groups with same letters indicate no statistical significance, and groups with different letters indicate significance at *p* value less than 0.05.

Higher oxidative stress leads to muscle atrophy and decreased myogenesis [36]. Hence, we measured three genetic markers that are related to oxidative stress. Among the three markers, FO decreased levels of pyruvate dehydrogenase kinase 4 (*Pdk4*), while increasing superoxide dismutase (*Sod2*) levels compared to the HF group in both males and females, with no differences in catalase (*Cat*). Sex differences were depicted, with female offspring overall having a higher relative expression of all genetic markers compared with male offspring. For *Cat*, as shown in Figure 8a,b, no significant differences were detected in both 8-week male and female offspring. Only sex differences were found, with female offspring of both LF and HF fathers having higher *Cat* expression compared to their respective male groups.

Another marker of oxidative stress, *Sod2* levels, was comparable between HF and LF in both female and male offspring. Interestingly, FO increased *Sod2* levels in both male and female offspring compared to their respective HF offspring groups (Figure 8d,e). Diet and sex differences were detected, with female FO offspring having higher expressions of *Sod2* compared to male offspring counterparts. Offspring of LF fathers had higher levels of *Sod2* compared to 8-week male LF offspring. FO demonstrated reduced levels of *Pdk4* in both male and female offspring compared to HF. No differences were observed between HF and LF in 8-week male offspring (Figure 8e), while HF increased levels of *Pdk4* in female offspring compared to LF (Figure 8f). Diet and sex differences were found: female offspring of LF- and HF-fed fathers showed a significant upregulation of *Pdk4* compared to male offspring counterparts (Supplemental Table S1). No differences in any of the markers were observed at 16 weeks (Supplemental Figure S3d–f).

## 4. Discussion

In this study, we investigated the impact of FO supplementation in fathers and its effect on the metabolic health of their offspring, specifically in the skeletal muscle. HF diet is known to induce insulin resistance and disrupt significant metabolic pathways. Consistent with this, we observed significant alterations in key metabolic pathways, including insulin signaling, oxidative stress, and fatty acid utilization, due to this diet. Interestingly, when paternal FO supplementation was introduced, we observed notable improvements in markers associated with insulin signaling, oxidative stress, muscle growth, and fatty acid utilization. To the best of our knowledge, this is one of the pioneering studies demonstrating the beneficial effects of FO supplementation in reducing paternal obesity and improving offspring skeletal muscle health.

An HF diet affects skeletal muscle function due to increased oxidative stress [37], which negatively affects insulin sensitivity [37,38]. One of the endogenous mechanisms of the body to combat oxidative stress is through activating the antioxidant pathway superoxide dismutase (SOD) pathways to decrease SOD production [37]. This was in line with our data, where FO increased *Sod2* compared to HF in both sexes. This sheds light on the beneficial effects of FO supplementation in the father, which improved antioxidant levels in the male and female offspring. This is similar to a study by Martins et al., which showed higher anti-oxidant potential with FO compared to HF supplementation [37]. In addition, they also found decreased H2 O2 production with FO compared to the HF group. Interestingly, we observed a similar *CAT* expression in the 8-week male FO group compared to other groups. A study conducted by Lanza et al. has demonstrated similar patterns with no difference in CAT levels between HF and FO [38], indicating that FO might not contribute to CAT expression [37]. However, specific mechanisms are not elucidated.

Another critical pathway altered with insulin resistance is insulin signaling. To measure insulin signaling, we measured players associated with insulin response: *Foxo1*, *Pi3k*, *Glut4*, as well as insulin sensitivity via ITT. We found that an HF diet caused insulin resistance compared to LF indicated by higher blood glucose values at 60 and 90 min with the ITT. Further, male offspring born to FO fathers had significantly lower blood glucose indicating better insulin sensitivity, as shown in Figure 3. Similar to our study, Martins et al. showed that FO supplementation improved muscle insulin responsiveness in adult male rats as evidenced by higher insulin sensitivity and a decreased homeostatic model assessment for insulin resistance (HOMO-IR) score compared to rats fed with HF exclusively [37]. Insulin activates *Irs1*, which causes a cascade of phosphorylation events, leading to the activation of *Pi3k*, *Akt*, and translocation of Glut 4 to the cell membrane. In our study, FO activated markers in the insulin signaling pathway as evidenced by an increased expression of *Glut4*, *Irs1,* and *Pi3k,* leading to a better insulin response in the offspring of FO-fed fathers compared to HF-fed fathers. In support of this evidence, studies by Lanza et al. found increased expression of *Glut4* and *Irs1* in the skeletal muscle of FO-fed adult male mice compared to HF [38]. Interestingly, all markers of insulin signaling were higher in female mice than their male counterparts. Additionally, we observed a better insulin response with a rapid decrease in blood glucose levels following insulin injection in females in ITT. This may be because the female mice have better insulin sensitivity compared to their male counterparts [39]. This could be responsible for the partial alleviation of skeletal muscle insulin resistance. 

Another defect with obesity is decreased mitochondria biogenesis, contributing to impaired mitochondrial dysfunction [13,14]. Further, many studies have found decreased mitochondrial size, number, and function with obesity [10,11,12,14]. Corroborating this, an HF diet is closely associated with mitochondrial dysfunction in skeletal muscle resulting from increased ROS production and an imbalance of mitochondrial fusion and fission. In addition, an HF diet induces fatty acid utilization in mitochondria and results in a massive production of ROS, thereby affecting mitochondrial structure and its cell membrane integrity in skeletal muscle [11,13,14]. A study found increased mitochondrial function with enhanced basal oxygen consumption and citric acid cycle intermediates in male adult mice supplemented with FO compared to mice fed HF. Our study found increased expression of *Mtco1* in female offspring, indicating a similar increase in mitochondrial function due to FO. 

Aside from that, another factor that contributes to insulin resistance is an accumulation of intramyocellular lipids among the insulin-resistant or T2D populations [32,40]. With an HF diet, a large influx of lipid enters the mitochondria, resulting in decreased rates of beta-oxidation and potential protein misfolding [41]. FO restores the balance of lipid synthesis and oxidation by reducing intracellular lipid levels by decreasing fatty acid synthesis and simultaneously upregulating beta oxidation via markers such as *Ppara* [42], which has proven to increase mitochondrial biogenesis. In line with the above studies, our study indicated decreased fatty acid synthesis as evidenced by decreased expression of *Fasn* and *Fabp4* and increased beta-oxidation with upregulation of *Ppara*, *Cidea*, and *Acat* with FO supplementation. Further, fatty acid synthesis was found to be higher in male mice compared to female mice. This might be because male C57BL6J mice are more sensitive to weight gain when exposed to an HF diet than female mice [43]. In line with this, Extier et al. also observed sex effects for FO metabolism with increased plasma and liver FO contents in female rats in contrast to males due to increased desaturase levels in females, resulting in higher fatty acid replenish rates [44]. Although our study exclusively looked at muscle tissue, the sex effects of FO still persist. 

Our study also highlights the beneficial effect of FO in the muscle as evidenced by increased expression of *Myoga* and *Fgf21*. Myog is essential for initiating myogenesis and also crucial for skeletal muscle recovery and regeneration in mice [45]. A recent study by Liu et al. demonstrated that FO alleviates muscle wasting [46]. Corroborating this, we observed increased *Myog* expression in both male and female offspring fed FO compared to HF supplementation. Another marker important for muscle growth is *Fgf21*, which was shown to protect against skeletal muscle wasting and weakness using fgf21 knockout mice [47]. In conformity with these findings, we found increased *Fgf21* expression in the FO offspring group compared to HF in both males and females. 

While most studies address the direct effects of FO on skeletal muscle health [16,17,46,48], the intergenerational effect of FO is not fully understood, especially consumption of FO during the preconception period by the father. Numerous studies showed that paternal obesity negatively affects offspring health [49,50,51]. One study reported low birth weight of progeny from obese rodent fathers with an imbalanced growth hormone/ insulin growth factor-I (IGF-1) ratio and abnormal muscle and fat development as evidenced by decreased expression of myogenic differentiation factor 1 and Igf1r expression, respectively [24]. On the other hand, paternal exercise endowed a protective effect of better insulin signaling to offspring when rodent fathers were supplemented with a high-fat diet [25,52]. Krout et al. have postulated that those progenies with an exercised HF father have a lower body fat percentage [25]. Specific mechanisms on how paternal obesity affects the offspring remain inconclusive, but several studies have pointed out epigenetics [53,54], which occurs through spermatozoa. Sperm is known to be affected by paternal obesity in terms of motility and mitochondrial activity. Research studies have indicated increased ROS in obese male spermatozoa damages its membranes and its content, which is transferred to the next generation [22]. The contents are transferred to the next generation through DNA methylation, histone modification, and (small non-coding RNA) sncRNAs. Among the three mechanisms, sncRNAs have been primarily studied with paternal obesity partially due to its abundance and diversity in sperm [55]. They play a critical role in regulating gene expression both transcriptionally and post-translationally [56]. In line with this, research has shown that sncRNA content in HF mice sperm contribute to metabolic dysfunction in offspring mice [56,57]. Further, paternal exercise conferred a protective effect of metabolic status of offspring with increased sperm quality as evid enced by a better sncRNA expression level [55]. Corroborating this, one study showed that overweight male sperm contained a lower methylation rate at certain gene regions [58]. On the other hand, the overall epigenetic mechanisms of sncRNAs remained elusive due to its origin [59]. 

Interestingly, predominant potential beneficial effects were observed in the offspring at 8 weeks compared to 16 weeks. It could be related to the developmental stages, where markers of insulin signaling and fatty acid synthesis were altered by FO at 8 weeks but not at 16 weeks. However, *Myog* and *Fgf21* expression were increased at 16 weeks, which are long-term muscle development markers. The profound impact in males we observed might be due to the male offspring’s relatively higher body weight, as pointed out by Du et al. [60]. It is possible that sex differences that we observed stemmed from differences in hormonal expression, such as estrogen, which plays a protective role in females. Estrogen in the form of 17-β estradiol (E2) is found to affect skeletal muscle glucose uptake [61,62,63], and a decreased level of E2 negatively affects insulin signaling in terms of decreased *Glut4* expression [62]. In line with this, research has indicated that incidence of T2D increased following menopause [64]. Further, E2 also improves lipid oxidation in skeletal muscle [63]. These studies used ovariectomized (OVX) female mice to highlight the importance of E2, and found disturbed insulin signaling pathways in terms of decreased expression and Ppara levels [62,63]. Collectively, we observed sex differences in the expression of the aforementioned genes as well. On the other hand, several studies also demonstrated the role of estrogen receptor α in insulin signaling [61,63]. A study by Marique et al. demonstrated the importance of estrogen receptor α to whole-body insulin resistance [65]. Increased body weight and decreased glucose uptake in soleus is observed in female estrogen receptor α (ER) knock-out mice [65], suggesting the protective role ER plays in maintaining whole-body insulin sensitivity.

While the results for this research indicate a potential role of fish oil in offspring skeletal and metabolism health, several limitations are noted. Immunoblotting would have provided validation of gene markers that we measured, which is lacking in the current study. Also, having a low-fat group as a control would have been ideal to determine health benefits of these supplements under healthy diets and in lean subjects. However, our primary goal was to determine the effects of fish oil in diet-induced obesity. Hence, such studies would be conducted in the future.

## 5. Conclusions

In conclusion, our study found that paternal supplementation of FO during the preconceptional period is more beneficial to the next generation, specifically in skeletal muscle. This study’s importance is that the father’s diet during the preconceptional period impacted their offspring’s metabolic health, when controlled for the mother’s diet. Such effects were observed at 8 and 16 weeks in the offspring, even when the offspring were fed a chow diet.

## Figures and Tables

**Table 1:** Distribution of offspring based on dietary groups and duration of study.

	LF	HF	FO	Total
Total offspring	41	65	67	173
Short term (ST; 8 weeks)				
Male	8	10	10	28
Female	10	8	10	28
Long term (LT; 16 weeks)				
Male	8	14	14	36
Female	10	13	13	36

**Table 2:** Primer sequences.

Gene	Forward	Reverse
*Acaca*	GCAGCAGTTACACCACATAC	TCCGCCATCTTCCACAATA
*Actin*	TGAGACCTTCAACACCCCAGCCA	CGTAGATGGGCACAGTGTGGGTG
*Cidea*	TCGGCTGTCTCAATGTCAA	GGATGGCTGCTCTTCTGTA
*Myoga*	CCTTCCTGTCCACCTTCA	CACCGACACAGACTTCCT
*Acat1*	ATATCTCACGGCAGGAACA	CACGCTTGTATTCTTCATCTTCT
*Fabp4*	CACCGAGATTTCCTTCAAACT	TATGATGCTCTTCACCTTCCT
*Fasn*	GTCGTCTATACCACTGCTTACT	ACACCACCTGAACCTGAG
*Glut4*	CAGTATGTTGCGGATGCTAT	TTAGGAAGGTGAAGATGAAGAAG
*Fgf21*	CCAAGACCAAGCAGGATTC	AGAGTCAGGACGCATAGC
*Irs1*	TCGCTAACTGAGATAGTCATACAA	TCCTGCTAACATCCACCTT
*PI3k*	GTCAGAAGGCAGGAGTCA	TAGAAGATGGCTTGGATGGAA
*Pdk4*	TGTGATGTGGTAGCAGTAGT	TGGTGAAGGTGTGAAGGA
*Foxo1*	GCTCTGTCCTGAAGAATCCT	CTAATCCTGCCACTGTCTGTA
*mt-Co1*	TCCCAGATATAGCATTCCCACG	ACTGTTCATCCTGTTCCTGC
*Cat*	AGCGACCAGATGAAGCAGTG	CCGCTCTCTGTCAAAGTGTG
*Sod2*	CAGACCTGCCTTACGACTATGG	CTCGGTGGCGTTGAGATTGTT
*PPAR-α*	TCGAGGAAGGCACTACACCT	TCTTCCCAAAGCTCCTTCAA

**Table 3:** Diet and sex difference across groups.

Gene Name	Sex (S)	Diet (D)	Interactions (S*D)
*Acat*	0.13	0.0019	0.0031
*Foxo 1*	<0.01	0.29	0.94
*Pdk4*	<0.01	<0.01	0.11
*CAT*	<0.01	0.08	0.0328
*Cidea*	<0.01	<0.01	0.48
*Fabp 4*	0.0214	<0.01	0.62
*Fasn*	<0.01	0.0251	<0.01
*Glut4*	0.0403	<0.01	0.0274
*Fgf21*	<0.01	<0.01	<0.01
*Irs 1*	<0.01	<0.01	<0.01
*Myog*	0.4625	<0.01	0.93
*Mtco-1*	<0.01	<0.01	<0.01
*Pi3k*	<0.01	<0.01	0.11
*PPAR alpha*	<0.01	<0.01	0.55
*SOD2*	<0.01	<0.01	0.36

## Data Availability

Data are contained within the article.

## Supplementary Materials

The following supporting information can be downloaded at: https://www.mdpi.com/article/10.3390/biomedicines11123120/s1, Figure S1: Gene expression of markers related to fatty acid oxidation and synthesis in muscle of 16-week male mice. Figure S2: Gene expression of markers related to insulin signaling in muscle of 16-week male mice. Figure S3: Gene expression of markers related to mitochondrial oxidation and oxidative stress in muscle of 16-week male mice. Table S1: Multiple comparisons between dietary groups of both sexes.
