# Supplementary material for: Role of Fish Oil in Preventing Paternal Obesity and Improving Offspring Skeletal Muscle Health
Source: Biomedicines. 2023 Nov 23;11(12):3120. doi: 10.3390/biomedicines11123120 (PMC10740802; doi:10.3390/biomedicines11123120)
Supplement: Supplementary file 1 [file biomedicines-11-03120-s001.zip › Supplemental Figures.pdf]

Supplemental Figure S1

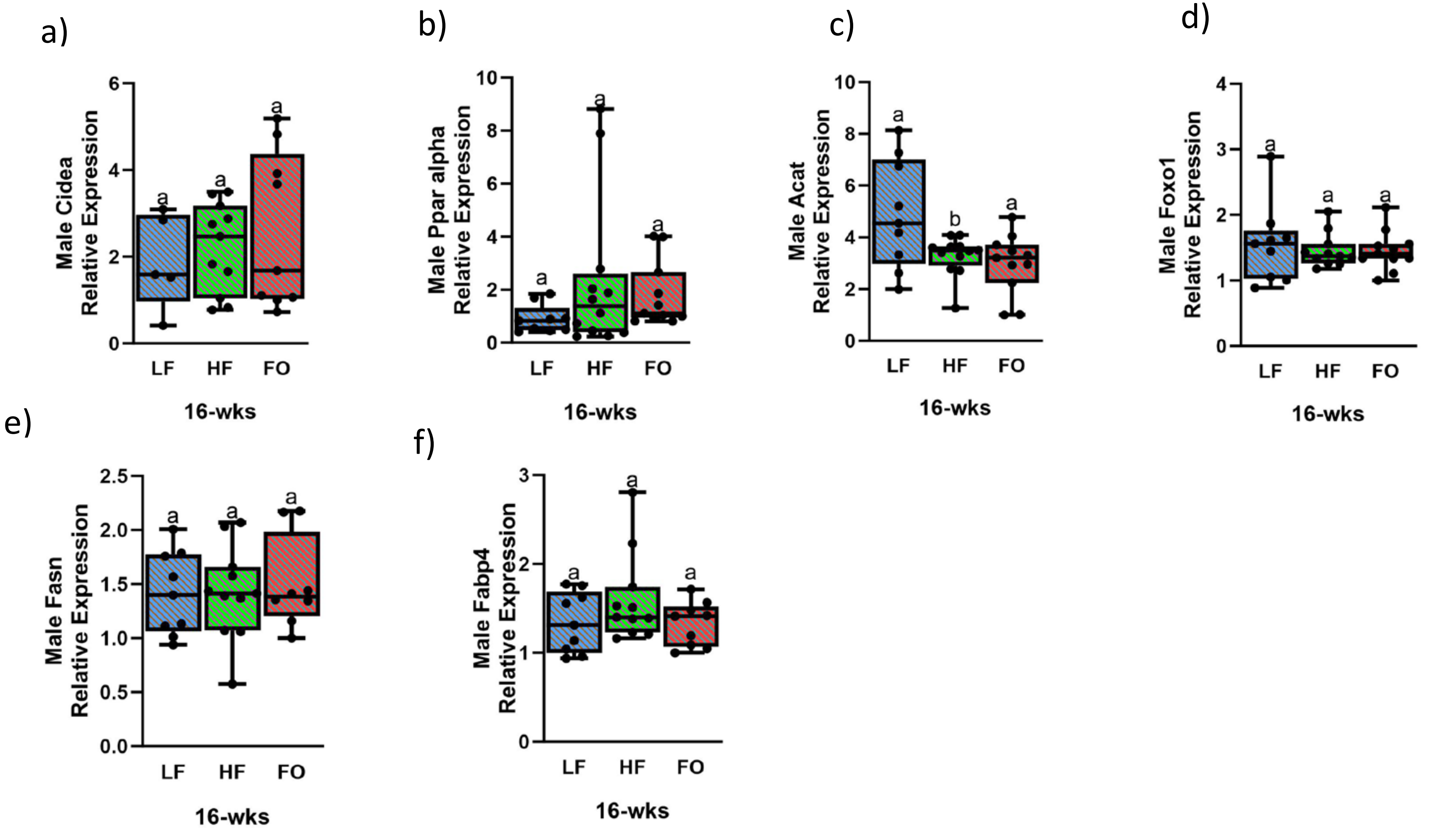

Figure S1: *Gene expression of markers related to fatty acid oxidation and synthesis in muscle of 16-week male mice. Relative normalized expression of (a) cell death-inducing DNA fragmentation factor (Cidea), (b) peroxisome proliferator-activated receptor alpha (Ppar alpha), (c) acetyl-CoA acetyltransferase (Acat), (d) forkhead box protein O1, (e) fatty acid synthase (Fasn), (f) fatty acid binding protein 4 (Fabp4). Common letters on the error bars indicate no significance (e.g., "a" is significantly different from "b" and "ab" indicates no significance compared to "a" and "b").*

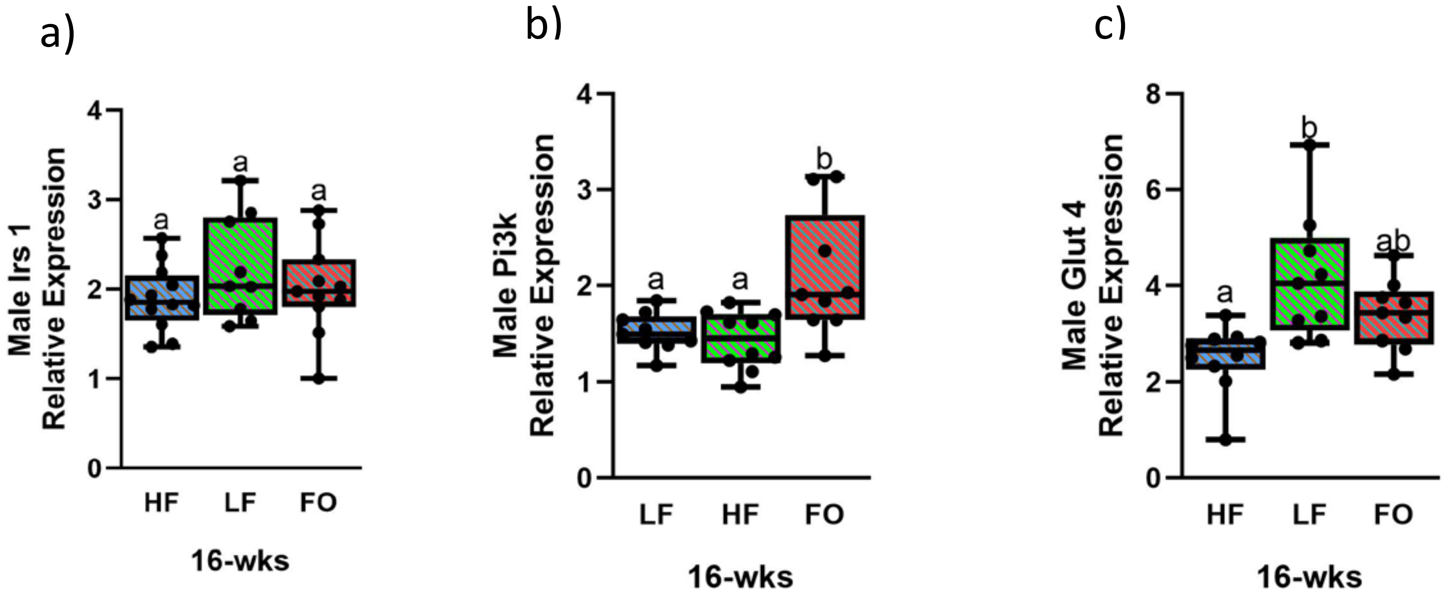

Figure S2: Gene expression of markers related to insulin signaling in muscle of 16-week male mice. Relative Normalized Expression of (a) glucose transporter type 4 (Glut4), (B) phosphatidylinositol-3 kinase (Pi3k), (c) insulin substrate 1 (Irs1). Groups with the same letter indicate no statistical significance and groups with different letters indicate significance at p value less than 0.05.

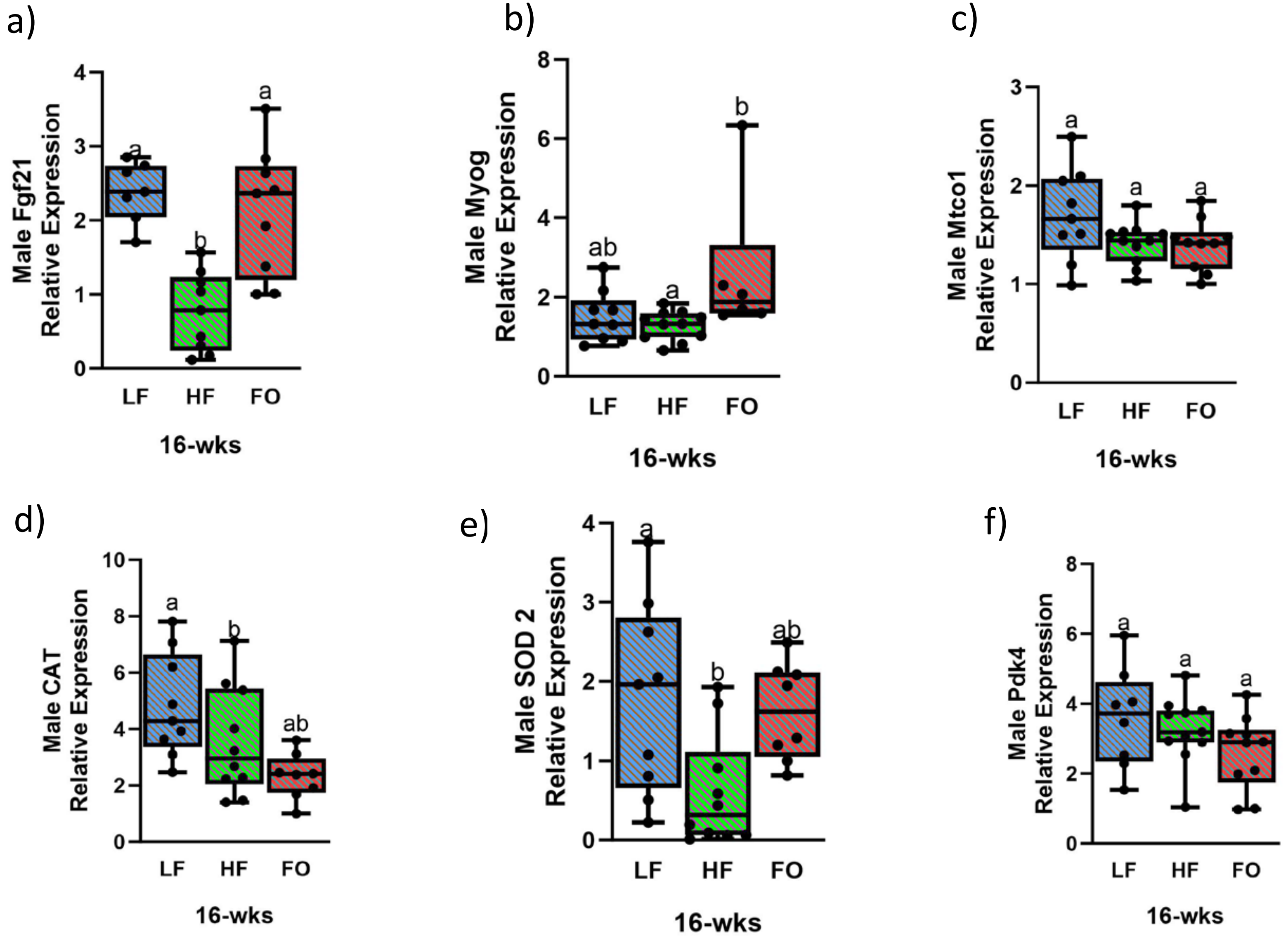

Figure S3: Gene expression of markers related to mitochondrial oxidation and oxidative stress in muscle of 16-week male mice. Relative normalized expression of (a) fibroblast growth factor 21 (Fgf21), (b) myogenin (Myog), (c) mitochondrially encoded cytochrome c oxidase I (mtco1), (d) catalase (CAT), (e) superoxide dismutase 2 (Sod2) and (f) pyruvate dehydrogenase kinase 4 (Pdk4).Groups with the same letters indicate no statistical significance and groups with different letters indicate significance at p value less than 0.05.
