# Supplementary material for: Role of Fish Oil in Preventing Paternal Obesity and Improving Offspring Skeletal Muscle Health
Source: Biomedicines. 2023 Nov 23;11(12):3120. doi: 10.3390/biomedicines11123120 (PMC10740802; doi:10.3390/biomedicines11123120)
Supplement: Supplementary file 1 [file biomedicines-11-03120-s001.zip › Supplemental table S1 Ray MS.pdf]

Supplemental Table S1: Multiple comparison of dietary groups between sexes

	Male-Female Multiple Comparisons at 8 weeks	
Acat	LF	0.21
	HF	0.6521
	FO	0.0050
Foxo 1	LF	0.0002
	HF	0.000027
	FO	0.0001
PDK 4	LF	0.000194
	HF	0.000027
	FO	0.000113
CAT	LF	0.0011
	HF	0.0002
	FO	0.6324
Cidea	LF	0.0674
	HF	0.3071
	FO	0.8675
Fabp 4	LF	0.7030
	HF	0.1223
	FO	0.6554
Fasn	LF	0.991
	HF	0.000005
	FO	0.30756
Glut 4	LF	0.9731
	HF	0.9079
	FO	0.0046
Fgf21	LF	0.2364
	HF	0.000017
	FO	0.9849
Irs 1	LF	0.5263
	HF	0.0053
	FO	0.00000005
Myog	LF	0.9993
	HF	0.8836
	FO	0.9316
Mtco-1	LF	0.8161
	HF	0.795
	FO	0.000018
Pi3k	LF	0.0050
	HF	0.0366
	FO	0.000009
PPAR alpha	LF	0.0016
	HF	0.0041

	FO	0.0574
Sod 2	LF	0.5263
	HF	0.7596
	FO	0.0255
